# Whole Blood Stimulation Assay as a Treatment Outcome Monitoring Tool for VL Patients in Ethiopia: A Pilot Evaluation

**DOI:** 10.1155/2020/8385672

**Published:** 2020-01-23

**Authors:** Yetemwork Aleka, Ana Victoria Ibarra-Meneses, Meseret Workineh, Fitsumbrhan Tajebe, Amare Kiflie, Mekibib Kassa Tessema, Roma Melkamu, Azeb Tadesse, Javier Moreno, Johan van Griensven, Eugenia Carrillo, Wim Adriaensen

**Affiliations:** ^1^Department of Immunology and Molecular Biology, University of Gondar, Gondar, Ethiopia; ^2^WHO Collaborating Centre for Leishmaniasis, National Centre for Microbiology, Instituto de Salud Carlos III, Majadahonda, Madrid, Spain; ^3^Leishmaniasis Research and Treatment Centre, University of Gondar, Gondar, Ethiopia; ^4^Unit of Neglected Tropical Diseases, Department of Clinical Sciences, Institute of Tropical Medicine, Antwerp, Belgium

## Abstract

Visceral leishmaniasis (VL) is a lethal disease if left untreated. Current treatments produce variable rates of treatment failure and toxicity without sterile cure, rendering treatment efficacy monitoring essential. To avoid repeated invasive tissue aspirates as well as empirical treatment, there is a need for new tools that allow a less-invasive and early assessment of treatment efficacy in the field. Cross-sectional studies have suggested levels of cytokines/chemokines after whole blood stimulation as good markers of cure, but longitudinal studies are lacking. In this study, we followed 13 active VL cases in an endemic area in Ethiopia by measuring the production of IFN-*γ*, TNF-*α*, IP-10, IL-2, IL-10, MCP-1, and MIG before, during, and at the end of treatment. After 24 hours of stimulation of whole blood with soluble *Leishmania* antigen, we observed an early, robust, and incremental increase of IFN-*γ*, TNF-*α*, and IP-10 levels in all patients during treatment. Moreover, based on the IFN-*γ* levels that showed an average 13-fold increase from the time of diagnosis until the end of treatment, we could almost perfectly discriminate active from cured status. Similar concentrations and patterns were found in stimulation assays with the two main *Leishmania* species. The levels of IFN-*γ*, IP-10, or TNF-*α* also seemed to be inversely associated with the parasite load at baseline. Despite a 1/10 drop in concentrations, similar patterns were observed in IFN-*γ* and IP-10 levels when dried plasma spots were stored at 4°C for an average of 225 days. All the above evidence suggests a detectable restoration of cell-mediated immunity in VL and its association with parasite clearance. With a potential application in rural settings by means of dried plasma spots, we recommend to further explore the early diagnostic value of such assays for treatment efficacy monitoring in large cohort studies including treatment failure cases.

## 1. Introduction

Visceral leishmaniasis (VL) is a neglected vector-borne protozoan disease, prevalent in the tropics, subtropics, and Mediterranean basin. It is responsible for an estimated 50,000-90,000 annual cases globally and lethal if left untreated [1, 2]. Over 90% of the cases are concentrated in Bangladesh, India, Brazil, Sudan, South Sudan, and Ethiopia [2]. It is characterized by persistent low-grade fever, weight loss, anemia, pancytopenia, hepatosplenomegaly, edema, muscle wasting, diarrhea, and hypergammaglobulinemia. Through time, it may cause bleeding due to thrombocytopenia which may progress to sepsis and severe cachexia [3, 4]. The limited range of drugs currently used for treatment includes pentavalent antimonials, pentamidine, amphotericin B deoxycholate and its lipid formulations, miltefosine, and paromomycin. However, they all produce considerable but variable rates of treatment failure (10-59%) and sterile cure is almost never achieved [5–10]. Due to the latter, the World Health Organization (WHO) defines “true cure” as a patient with no relapse episode in 3-6 months after treatment stop. In addition, the treatments are lengthy, have a high toxicity with regular side effects, and are often expensive [11]. Taken together, the need to monitor drug efficacy is paramount.

Considering the lack of comparable alternatives, WHO recommends a splenic, bone marrow or lymph node aspirate as a test for diagnosis but also as a test of cure [12]. Nevertheless, patients are often discharged purely following improvement of clinical signs in more rural settings, due to the need of an experienced health worker to perform the procedure or bleeding risk. To avoid repeated invasive tissue aspirates as well as empirical treatment, there is a need for quick, easy-to-use, and sensitive tools that allow a less-invasive assessment of treatment efficacy. Such tools could aid clinicians in making decisions about the continuation or change of treatment regimens. Moreover, markers able to identify the initiation of a successful treatment might help to shorten the treatment duration.

Serological techniques cannot be used for this purpose as antibodies remain high for several years. Molecular techniques seem promising, but could be less suitable as the parasitic load mostly decreases steeply after two days of treatment and gives no information on the host's immunological recovery [13–15]. Following the latter, one of the key immunological characteristics of active disease is a profound immunosuppression and impaired production of interferon-*γ* (IFN-*γ*) and associated cytokines. Activation of infected macrophages by IFN-*γ* triggers the production of nitric *oxide synthases*/reactive oxygen species (NOS/ROS) that facilitates parasite killing, indicating the importance of cellular sources of IFN-*γ* [16, 17]. The host's *Leishmania*-specific cell-mediated immunity is thus a key determinant in resistance and clearance of a *Leishmania* infection and could serve as a proxy measurement of the host's capacity to maintain future recrudescence or to resist reinfections of *Leishmania* as it is known to last for several years [18]. Therefore, techniques such as the whole blood stimulation assay (WBA) which measures the *in vitro* cell-mediated immune response after stimulation with soluble *Leishmania* antigens have been proposed to monitor disease recovery [19, 20]. Recent findings in cross-sectional studies of Ethiopia, Spain, and Bangladesh further enforced the idea that gradual increases in IFN-*γ* or in IFN-*γ*-induced protein 10 (IP-10 or CXCL10), monokine induced by IFN-*γ* (MIG or CXCL9), tumor necrosis factor-alpha (TNF-*α*), and monocyte chemoattractant protein 1 (MCP-1 or CCL2) levels in soluble *Leishmania* antigen- (SLA-) stimulated whole blood could indicate that treatment has been successful and could be targeted as biomarkers of clinical cure in VL [20–23]. The search for a robust marker is still ongoing, as different cytokines and chemokines have been proposed, but vary by geographical area, *Leishmania* strain, and outcome identification (asymptomatic, active, cured). To date, this assay has never been evaluated longitudinally to monitor individual *L. donovani*-infected patients during the course of treatment. And only one cross-sectional study reported on its value in the East African *L. donovani* setting, which was restricted to the IFN-*γ* and interleukin- (IL-) 10 analytes. In remote regions, a simpler storage and transportation method would be highly needed. Protein Saver 903 cards have shown potential as an alternative method for transporting SLA-stimulated plasma samples, even at ambient temperature, for the later analysis of IL-2, IFN-*γ*, IP-10, MIG, and MCP-1 concentrations [24].

The main aim of this pilot study was to further validate the usefulness of this assay as a treatment efficacy monitoring tool in Ethiopian VL patients by means of a first-in-its-kind longitudinal evaluation of individual patients during the course of treatment. To further fine-tune the development and implementation of a WBA in research and clinical activities among VL patients in East Africa, we also assessed strain specificity, a more field-adapted method, and demonstrated the value of seven previously proposed cytokine/chemokine markers.

## 2. Materials and Methods

### 2.1. Study Design

An institutional-based cohort study was conducted from March to September 2018 in which a total of 21 VL patients were recruited at time of diagnosis (referred to as day zero (D0)). A 2 ml venous blood sample was collected in a lithium-heparin tube at D0, after the first week of the treatment (W1), and at end of treatment (EOT). Sociodemographic data was collected by questionnaire and clinical data was collected from the patient's medical record by using a case report format. One patient died during treatment because he suffered from acute kidney injury and anemia. In addition, three were lost to follow-up during treatment and four had a missing time point. Therefore, a total of 13 VL patients with complete follow-up during treatment was included in the main analyses. A total of 19 VL patients were used for baseline analyses.

The study protocol was reviewed and approved by the research ethics committee of the School of Biomedical and Laboratory Sciences, College of Medicine and Health Sciences, University of Gondar, and the Institutional Review Board of the Institute of Tropical Medicine. All study participants provided informed consent.

### 2.2. Study Population

Patients were recruited and followed at the Leishmaniasis Research and Treatment Centre (LRTC) at the University of Gondar, Ethiopia. The center is located in the northwestern part of Ethiopia close to the high VL-endemic areas along the border with Sudan. Only microscopically confirmed VL cases (by the spleen or bone marrow aspiration) were included in the study. Patients with a concurrent medical emergency (unconscious and severely ill individuals), documented coinfection (tuberculosis (TB), human immunodeficiency virus (HIV), and helminthic infection), records of any immune suppressive drugs, or who were younger than 18 or older than 65 years were considered noneligible.

### 2.3. Operational Definitions

Body mass index (BMI) was defined as the weight in kilograms divided by the square of the height in meters (kg/m^2^), and the following criteria were used: underweight (BMI < 18.5 kg/m^2^) and normal or healthy weight (BMI within the range of 18.5-25 kg/m^2^) [25]. Fever was considered if the body temperature was above 37°C.

Cure from leishmaniasis was defined at clinical discharge based on the absence of clinical signs or if a repeated splenic or bone marrow aspirate was taken, negative microscopy at end of treatment [26]. Following WHO guidelines, seven of the 13 VL patients (54%) confirmed “true cure” status at six months after therapy and six patients were lost to follow-up.

After splenic/bone marrow aspiration, parasites were detected with standard Giemsa staining. The parasite density score was determined using a logarithmic scale ranging from 0 (no parasites per 1000 oil immersion fields), 1+ (1–10 parasites per 1000 fields), 2+ (1–10 parasites per 100 fields), 3+ (1–10 parasites per 10 fields), 4+ (1–10 parasites per field), 5+ (10–100 parasites per field) to +6 (>100 parasites per field) [27].

### 2.4. Preparation of Soluble *Leishmania* Antigen (SLA)

*L. donovani* and *L. infantum* antigens were prepared from promastigote cultures in the stationary phase (S-698 strain MHOM/ET/67/HU3 and JPC strain MCAN/ES/98/LLM-722, respectively) as previously described [28, 29]. The parasites were first washed with 1X phosphate-buffered saline (PBS) and centrifuged at 1000 g for 20 min at 4°C. The supernatant was discarded, and the pellet resuspended in lysis buffer (50 mM Tris/5 mM EDTA/HCl, pH 7). These samples were subjected to three cycles of freezing/thawing and then sonicated three times (40 W for 20 s) before being centrifuged again at 27,000 g for 20 min at 4°C. The supernatants were collected and centrifuged at 100,000 g for 4 hours at 4°C, to remove the membrane antigens. Finally, the supernatants were divided into aliquots and stored at -80°C until use. The protein content was quantified following the bicinchoninic acid method (BCA), using the Pierce BCA Protein Assay Kit (Bio-Rad, USA).

### 2.5. Whole Blood Stimulation Assay (WBA)

Whole blood samples were stimulated as previously described [20]. Briefly, for each sample, an aliquot of 500 *μ*l blood was transferred in a control tube (unstimulated), two tubes containing 10 *μ*g/ml SLA from *L. donovani* and 10 *μ*g/ml SLA from *L. infantum*, and a positive control tube with phytohemagglutinin (PHA). The latter and SLA of both species were lyophilized for utility and preservation in the field. Next, all of tubes were incubated at 37°C for 24 h. After incubation time, the supernatants were collected and stored at -80°C until cytokine/chemokine determination.

### 2.6. Storage of Stimulated Plasma Samples in Filter Paper

In parallel with freezing down stimulated plasma, 40 *μ*l was dropped onto two premarked 1.2 cm diameter circles on separate Protein Saver 903 cards (Whatman, Maidstone, UK) and dried for 3–4 h at ambient temperature (AT) in a horizontal position on the bench to produce dried plasma spots (DPS). The cards were then placed in zip-lock plastic bags (Whatman, Maidstone, UK) containing a desiccant and maintained at 4°C up to 8 months.

After this period, a biopsy punch (Integra Miltex, NY, USA) was used to cut out two discs from the cards of each patient. The discs were placed in the 96-well plate and 70 *μ*l of the elution buffer (2% BSA, 0.1% Tween-20 in PBS 1X) was added. The plate was incubated at ambient temperature for 1 hour in a shaker to elute all cytokines and chemokines of plasma deposited on filter paper.

### 2.7. Cytometric Bead Array (CBA) Assay

Levels of IFN-*γ*, TNF-*α*, IP-10, IL-2, IL-10, MIG, and MCP-1 were quantified in frozen plasma and DPS-elucidated sample from control and SLA-stimulated and PHA-stimulated whole blood using the BD Cytometric Bead Array Human Flex Set (Becton Dickinson Biosciences, USA) following the manufacturer's instructions. Briefly, 50 *μ*l of the plasma of each patient was incubated for 1 h at ambient temperature with 50 *μ*l of capture beads. After incubation, 50 *μ*l of the detection antibody was added and the mixture was placed 2 hours at ambient temperature. Data were acquired using a FACSCalibur flow cytometer and analysed using the Flow Cytometric Analysis Program Array (BD Biosciences, USA) by manual clustering. Cytokines and chemokine concentrations (expressed in pg/ml) produced in response to SLA or PHA stimulation were determined by subtracting background levels measured in the negative control samples (nonstimulated tube).

### 2.8. Data Analysis

**Figure 1:**
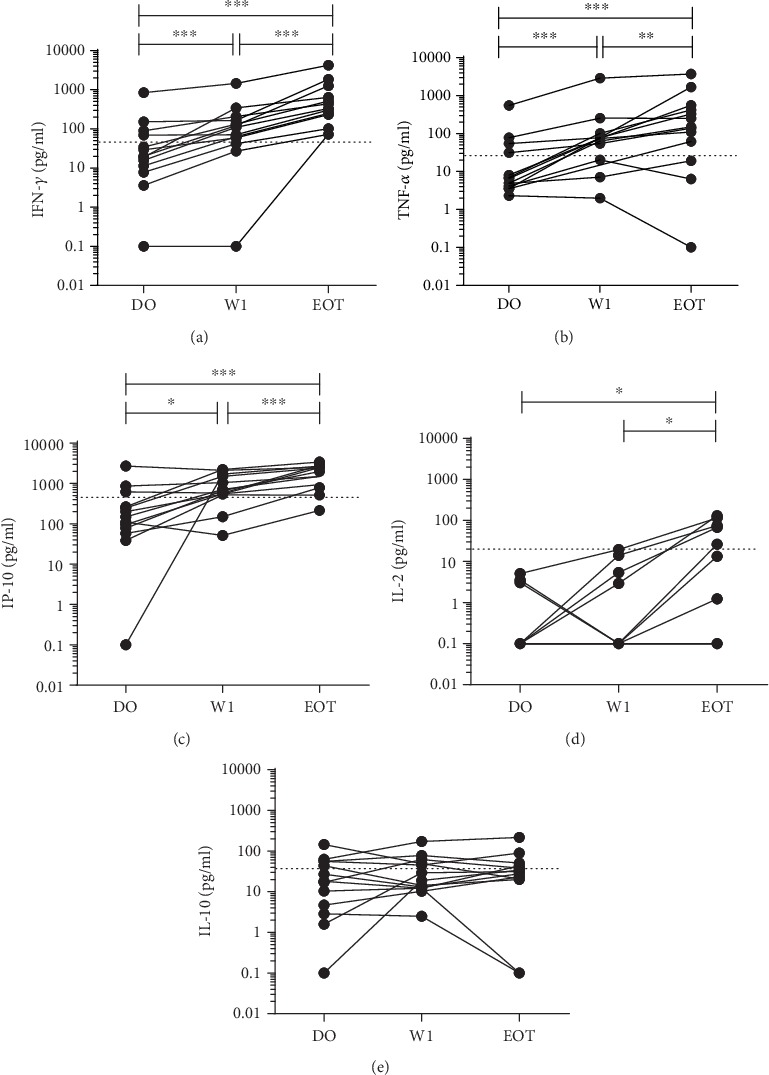
IFN-*γ* (a), TNF-*α* (b), IP-10 (c), IL-2 (d), and IL-10 (e) levels in soluble *L. donovani* antigen-stimulated plasma of 13 VL patients at active disease (D0), after one week of treatment (W1), and at the end of treatment (EOT). Each line curve represents an individual during follow-up. Comparison of medians was made using the Wilcoxon paired *t*-test. ^∗^*p* < 0.05; ^∗∗^*p* < 0.01; ^∗∗∗^*p* < 0.001. Dotted line represents the best cut-off for each cytokine/chemokine based on ROC analyses to discriminate active from cured status.

**Figure 2:**
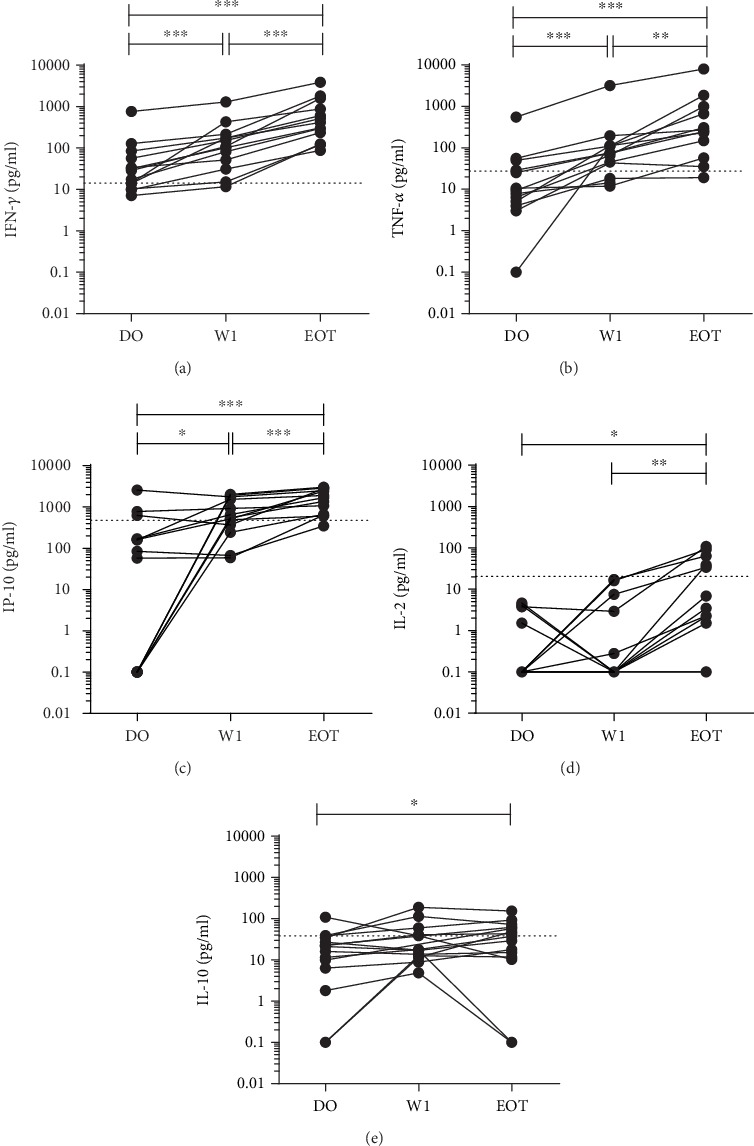
IFN-*γ* (a), TNF-*α* (b), IP-10 (c), IL-2 (d), and IL-10 (e) levels in soluble *L. infantum* antigen-stimulated plasma of 13 VL patients at the active moment (D0), during one week of treatment (W1), and the end of treatment (EOT). Each line curve represents an individual during follow-up. Comparison of medians was made using the Wilcoxon paired *t*-test. ^∗^*p* < 0.05; ^∗∗^*p* < 0.01; ^∗∗∗^*p* < 0.001. Dotted lines represent the best cut-off for each cytokine/chemokine based on ROC analyses to discriminate the active from cured status.

Data analysis was performed with GraphPad Prism v6.0 software (GraphPad Software, USA). Continuous data was presented as the median with interquartile ranges (IQR) and the normality of data was checked by the Shapiro-Wilk test. The concentrations of analytes before and after treatment were compared using the nonparametric Wilcoxon signed-rank test. The cut-off values for each cytokine and chemokine shown in Figures 1 and 2 were determined by calculating the optimal point in the receiver operating characteristic (ROC) curves for discriminating active disease from cured status. Spearman correlation coefficients were calculated between baseline parasite load and cytokine/chemokine concentrations or *L. infantum*- and *L. donovani*-stimulated cytokine levels. A *p* value ≤ 0.05 was considered as statistically significant.

## 3. Results

### 3.1. Sociodemographic and Clinical Characteristics of VL Patients

A total of 13 confirmed primary VL cases were included. All the patients were male with a median age of 22 years and only five (38.4%) were literate. Almost all (92.31%) were migrant workers, functioning as farmers or daily labourers (see Table 1). Related to their clinical condition, the median body mass index (BMI) was 16.4 kg/m^2^, classifying 12 (92.3%) patients as being underweight. Other pathologies were diagnosed in three (23.1%) patients, such as pneumonia (*n* = 1), malaria (*n* = 1), and giardia (*n* = 1). Regarding the treatment of leishmaniasis, 10 (76.9%) of them were treated with a combination treatment of paromomycin (PM) and sodium stibogluconate (SSG) while three patients (38.5%) were treated with AmBisome monotherapy. All patients were successfully treated and discharged.

### 3.2. IFN-*γ*, TNF-*α*, and IP-10 in SLA-Stimulated Plasma as Potential Biomarkers for Treatment Efficacy Monitoring in VL Patients

The cytokine/chemokine concentrations were measured in the soluble *L. donovani* antigen-stimulated plasma of VL patients at time of diagnosis (D0), during treatment (W1), and at the end of treatment (EOT) (Figure 1). The levels of IFN-*γ*, TNF-*α*, and IP-10 increased significantly after one week of treatment (median (IQR; *p* value) = 110.70 pg/ml (50.81-187.30; <0.01); 74.63 pg/ml (28.72-95.61; <0.01); and 667.90 pg/ml (533.80-1601; 0.01), respectively) compared to a baseline levels (D0) (median (IQR) = 19.96 pg/ml (9.62–79.85); 6.56 pg/ml (3.86–43.25); and 149.40 pg/ml (68.01-440.90), respectively). The levels of these cytokines/chemokines also presented a significant increase at the end of treatment with a dose-response like relationship (median (IQR; *p* value) = IFN-*γ*: 331.7 pg/ml (167.80-950.80; <0.01); TNF-*α*: 150.60 pg/ml (40.68-489.30; <0.01); and IP-10: 1979 pg/ml (863.30-2603; <0.01)) (Figures 1(a)–1(c)). The concentration of IL-2 was not significantly increased at W1 (median (IQR; *p* value) = 0.1 pg/ml (0.10-4.16; <0.5)), but significantly increased at EOT (median (IQR; *p* value) = 1.24 pg/ml (0.10-71.57; <0.05)), compared to D0 (0.1 pg/ml) (Figure 1(d)). IL-10 did not show significant differences between the different time points (Figure 1(e)). MCP-1 and MIG concentrations were lacking a defined pattern across the different time points of the study (data not shown).

To assess the specificity against the *L. donovani* strain used in the assay, we performed a simultaneous stimulation with the soluble *L. infantum* antigen. We found that the stimulation with SLA from *L. infantum* showed the same pattern of cytokine and chemokine production than stimulation with SLA from *L. donovani* (Figure 2). The results show a significant increase in IFN-*γ*, TNF-*α*, and IP-10 after one week of treatment (W1, median (IQR; *p* value) = 108.9 pg/ml (41.34-194.50; <0.001); 74.38 pg/ml (30.81-111.70; <0.001); and 552.10 pg/ml (306.00-1659; <0.05)) compared to baseline levels (D0) of IFN-*γ*, TNF-*α*, and IP-10 (median (IQR) = 28.64 pg/ml (12.15-70.48); 9.38 pg/ml (4.54-39.40); and 84.81 pg/ml (0.10-399.50), respectively). The levels of these cytokines/chemokines also presented a significant increase at the end of treatment compared to D0 (EOT, median (IQR; *p* value) = IFN − *γ* : 361.20 pg/ml (108.20-1221; <0.001); TNF-*α*: 247.50 pg/ml (80.12-908.60; <0.001); and IP-10: 1284 pg/ml (651.00-2646; <0.001)) (Figures 2(a)–2(c)). IL-2, IL-10, MIG, and MCP-1 also showed very similar patterns as previously described using the *L. donovani* antigen (Figures 2(d)–2(e)). Moreover, excellent correlation scores were observed for IFN-*γ* (*r* = 0.97; *p* < 0.001), TNF-*α* (*r* = 0.95; *p* < 0.001), and IP-10 (*r* = 0.97; *p* < 0.001) levels with *L. infantum* and *L. donovani* antigen stimulation. We also observed good correlations for IL-2 (*r* = 0.78; *p* < 0.001), IL-10 (*r* = 0.84; *p* < 0.001), MIG (*r* = 0.86; *p* < 0.001), and MCP-1 (*r* = 0.74; *p* < 0.001) levels with *L. infantum* and *L. donovani* antigen stimulation.

Comparative results were also obtained in response to the general T cell mitogen PHA, which showed a low production of cytokines and chemokines before treatment that increased during treatment (see in Table S1 in Supplementary Material). This indicated a good cell viability and immunocompetence after cell activation for all samples, but also confirmed the overall hyporesponsiveness observed during active VL disease and its restoration upon successful treatment.

### 3.3. IFN-*γ* Showed the Highest Fold Increase after SLA Stimulation Plasma at the End of Treatment

**Figure 3:**
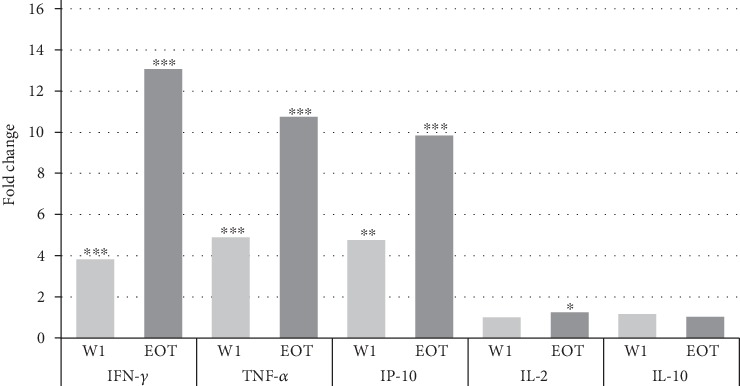
Fold changes in cytokine/chemokine concentrations during treatment of 13 VL patients, measured in soluble *L. donovani* antigen-stimulated plasma. Fold increase during W1 and EOT compared to D0 was calculated by dividing the value of (W1, EOT) by D0 values. *p* values are represented for comparison with baseline concentration at the time of diagnosis (D0). ^∗^*p* < 0.05; ^∗∗^*p* < 0.01; ^∗∗∗^*p* < 0.001.

The fold change in each cytokine/chemokine concentration after one week of treatment (W1) and at the end of the treatment (EOT), in relation to their concentration at the time of active disease (D0), is shown in Figure 3. The concentration of IFN-*γ* (*p* < 0.001) in the SLA-stimulated plasma increased 3.8 times after one week of treatment, while TNF-*α* (*p* = 0.001) and IP-10 (*p* = 0.013) increased around fivefold (Figure 3). At the end of the treatment, the concentrations of IFN-*γ* (*p* < 0.001), TNF-*α* (*p* < 0.001), and IP-10 (*p* < 0.001) increased significantly by 13.1, 10.8, and 9.9 times, respectively. IL-2 levels were also increased 1.3 times from D0 to EOT in a statistically significant manner (*p* = 0.023). In addition, we found very similar fold changes using the soluble *L. infantum* antigen as specific stimulation (see Figure S1 in Supplementary Material).

Without exception, all 13 patients experienced a steep and incremental increase during treatment in IFN-*γ* and TNF-*α* levels towards successful disease recovery, arguing to investigate its prognostic value in larger cohorts including treatment failures as a potential pretest-posttest.

### 3.4. IFN-*γ* Levels Showed Highest Discriminatory Power to Determine a Cured Status in VL Patients at End of Treatment

To investigate the possibility of a general, single-time point-based test instead of a pre-post treatment evaluation, we calculated the optimal cut-off values to determine a cured (EOT) status by means of ROC analyses. For IFN-*γ*, TNF-*α*, IP-10, IL-2, and IL-10 with *L. donovani* stimulation, the optimal cut-off values were 48.13 pg/ml, 25.35 pg/ml, 452.90 pg/ml, 20.02 pg/ml, and 37.29 pg/ml, respectively (depicted as dotted lines in Figures 1 and 2).  Table 2 shows the proportion of patients that produced levels above the respective cut-off values after SLA stimulation. Results showed that active VL patients recovered their ability to mount a specific cellular immune response against *Leishmania* during the treatment period, producing IFN-*γ*, TNF-*α*, IP-10, and IL-2 (with the exception of IL-10). IFN-*γ*, TNF-*α*, and IP-10 seemed to be the most promising biomarkers after one week of treatment. At end of treatment, a production of IFN-*γ* and IP-10 above the cut-off was able to identify 100% and 92% of the cured patients, arguing for further research towards a universal cut-off value to define cure. Yet, 3-4 VL patients did produce cytokine levels above the cut-off value at D0 (see Table 2), arguing for further optimization towards a universal cut-off value to define cure. These 4 patients all showed a median increase of 5.0, 1.7, and 7.6 times at EOT for IFN-*γ*, TNF-*α*, and IP-10, respectively.

### 3.5. IFN-*γ* and IP-10 Are Stable Markers in Filter Paper

**Figure 4:**
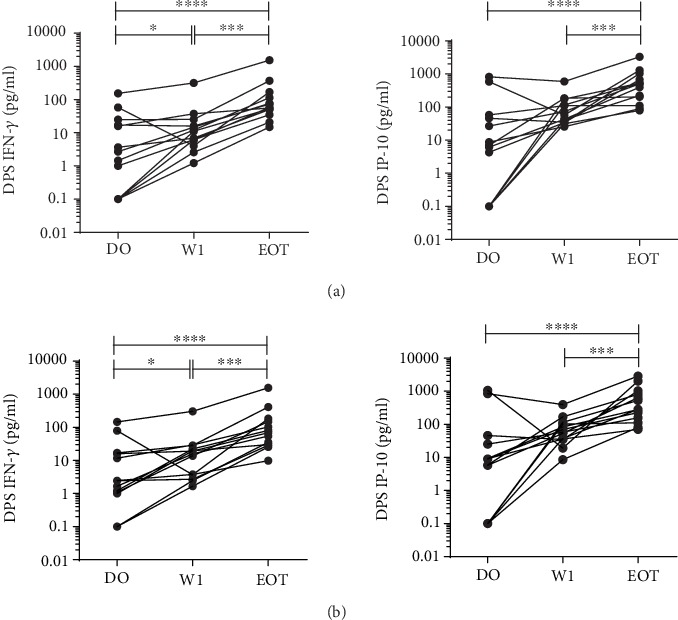
Levels of IFN-*γ* and IP-10 eluted from dried plasma spots (DPS) stored at 4°C for a median of 255 days in VL patients at D0, W1, and EOT. Blood was stimulated with lyophilized (a) *L. donovani* antigen or (b) *L. infantum* antigen. Each line curve represents one individual. The Wilcoxon test was used to compare paired samples. ^∗^*p* < 0.05; ^∗∗∗^*p* < 0.001; ^∗∗∗∗^*p* < 0.0001.

After elution of the SLA-stimulated plasma (*L. donovani* or *L. infantum* antigen) from the filter paper (DPS) that was stored at 4 °C for a median of 225 (IQR: 106-266) days and shipped at ambient temperature, we found that IFN-*γ* and IP-10 were the most stable analytes (Figure 4). It is important to highlight that the levels of TNF-*α*, IL-2, and IL-10 after elution of filter paper were below the detection limit of the technique, and that MIG and MCP-1 values after elution of the filter paper were also on the low side and, in comparison with frozen plasma, lacking a defined pattern across the different time points of the study. Compared to the frozen plasma method, concentrations were around 1/10 lower, indicating a significant loss of material due to the nature of the preservation method, as previously described [24]. Nevertheless, we could still observe similar patterns and IFN-*γ* and IP-10 levels significantly increased after one week of treatment with soluble *L. donovani* stimulation (median (IQR; *p* value) = 11.22 pg/ml (5.10-20.68; 0.0327) and 57.23 pg/ml (34.47-144.50; 0.1909), respectively) compared to baseline levels (D0). The levels of these cytokines/chemokines also presented a statistically significant increase at the end of treatment compared to D0 (median (IRQ; *p* value) = 58.37 pg/ml (42.7-141.9; 0.0002) and 501.4 pg/ml (156.50-855; 0.0002), respectively) (Figure 4(a)).

With *L. infantum* stimulation, we observed similar differences (Figure 4(b)). These findings indicate the stability of the patterns of the IFN-*γ* and IP-10 in a more field-adapted preservation method and robustness of the findings.

### 3.6. IP-10, TNF-*α*, and IFN-*γ* Concentrations Showed Significant Correlation with the Parasite Load at Baseline

To assess whether produced cytokine/chemokine levels in stimulated blood are reflecting parasite reduction in the spleen, we studied the correlation between spleen baseline parasite load and SLA-stimulated responses in whole blood.

**Figure 5:**
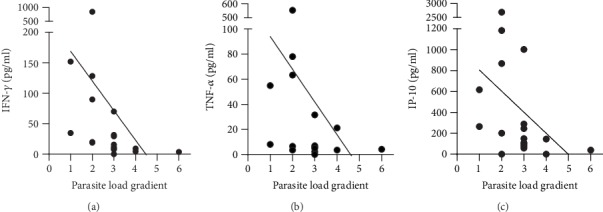
Correlation of baseline parasite load with IFN-*γ* (a), TNF-*α* (b), and IP-10 (c) levels after SLA *L. donovani* stimulation at D0. Correlation between analyte concentrations with parasitic load was based on all active VL cases of which baseline data was available (*n* = 19), by means of the nonparametric Spearman test.

Of all cytokines tested, an inverse correlation between the baseline concentration of IFN-*γ* (*r* = −0.74, *p* < 0.001), TNF-*α* (*r* = −0.52, *p* = 0.022), or IP-10 (*r* = −0.56, *p* = 0.012) and the baseline parasite load could be observed (Figure 5). This inverse relationship suggests that a recovery in cytokine/chemokine production after SLA stimulation could be used as a proxy for parasite reduction at end of treatment.

## 4. Discussion

In this small but unique cohort study of primary VL cases, 13 Ethiopian patients with successful treatment all showed a steep and incremental restoration of cellular IFN-*γ* production upon *in vitro* stimulation with soluble *Leishmania* antigens during treatment. This reflected a detectable restoration of the profound immunosuppression previously reported in active VL patients [30]. Despite being in contrast with a previous cross-sectional study in India where no statistically significant differences were found after quantification of IFN-*γ* levels between active and cured VL patients [31], our results consolidated the findings of the sole East African report. The latter reported on an incremental increase in IFN-*γ* levels in cured individuals living in Northwest Ethiopia as compared to active patients but could not detect differences in IL-10 levels. Similar findings were reported in Turkish, Indian, Iranian, Spanish, and Bangladeshi VL patients [20, 21, 23, 32, 33], indicating a typical cellular hyporesponsiveness during active disease that restores after successful therapy, as was also reported in studies using peripheral blood mononuclear cell (PBMC). This was also reflected in the gradual increase in nonspecific PHA stimulation which suggests a broad mechanism of immunosuppression that merits further investigation. Studies in Spain and Ethiopia demonstrated that a long-lasting memory response remains in cured patients and increased levels of IFN-*γ* and IFN-*γ*-inducible protein 10 (IP-10) could be detected in almost all patients at 6 and 12 months after treatment [21, 23]. This is, however, the first time that a longitudinal study in diseased individuals was performed with an early measurement at one week of treatment. It showed an early almost dose-response-like rate of recovery with 9 to 13-fold increases in IFN-*γ*, IP-10, or TNF-*α* levels after therapy (EOT).

Due to the sharp 13-fold increase in IFN-*γ* levels during treatment, it was almost possible to perfectly discriminate between diseased (D0) and cured (EOT) status. Although we did not have splenic parasite counts at end of treatment, the levels of IFN-*γ*, IP-10, or TNF-*α* seemed to be inversely associated with the parasite load at baseline and therefore could reflect parasite clearance. This further argues for the development and validation of this assay as an alternative test of cure in larger cohort studies including treatment failure cases to establish its prognostic and diagnostic value in early to late stages of the treatment.

Because IFN-*γ* is produced in rather low quantities, other IFN-*γ*-associated cytokines and chemokines have been proposed to show better performance. The production of IP-10 has a role in the host response to VL by initiation of early immune responses and further increasing the production of IFN-*γ*, which indirectly promotes killing of the intracellular parasite [34]. In our study, plasma concentrations of IP-10 after SLA stimulation were also significantly increased during successful treatment, but not as robustly in all individual patients as IFN-*γ* or TNF-*α*. A previous study also showed IP-10 as an accurate global marker of infection and cure [21]. We found similar results for TNF-*α* levels during treatment. TNF-*α* has an indirect role for parasite clearance by stimulating the production of IFN-*γ*. Previously, a significantly elevated production of TNF-*α* was also observed in VL-cured solid organ transplant subjects and VL-cured individuals from Spain after 6 months of treatment [20, 35].

Even though Th1 cell-mediated immunity for parasite control is associated with production of IL-2 by activated T cells [16], it was produced at much lower concentration and there was no significant production of IL-2 during treatment but only at the end of treatment. In previous studies, its concentration was also below detection limit during active infection while cured individuals were able to produce a significant concentration of this cytokine [20, 21]. This suggests that production of IL-2 is rather a late signature of cure.

High levels of the anti-inflammatory cytokine IL-10 have been associated with the depressed cell-mediated immunity (CMI) during active VL, and PBMC-based assays have showed elevated production of this cytokine during active infections that decreased over time [20, 36]. Nevertheless, we observed no changes in IL-10 levels during treatment which was also reported by Adem et al. in a very similar patient population [23]. These challenge the common dogma in PBMC assays or point towards other sources of IL-10. In a similar manner, our findings showed no specific pattern for MIG levels during treatment with SLA or PHA stimulation, although a previous study in Spain reported MIG to be a good biomarker of cure in *L. infantum-*infected individuals [21].

A similar whole blood interferon-*γ* release assay is being used in tuberculosis patients to identify specific immunity during latent infection (commercialized as QuantiFERON®) [37]. The *Leishmania*-specific whole blood stimulation assay has also been evaluated in different endemic areas of *Leishmania* spp. for the detection of subjects with asymptomatic infection [36, 38, 39]. However, specificity problems of the soluble *Leishmania* antigens used, in particular cross-reactivity with similar pathogens like *Trypanosoma cruzi* and *Toxoplasma gondii*, could be problematic when targeting a diagnostic or predictive tool in coendemic countries, which may be of lesser concern for its development as a test-of-cure. Field applicability is also important when testing for asymptomatic infection in the rural settings of endemic areas. We found comparable patterns in cytokine/chemokine expressions of frozen plasma and dried plasma spots that were stored at 4°C for up to 8 months and shipped at ambient temperature. Similar results were also obtained on negative control subjects and asymptomatic individuals from Spain and Bangladesh, where the samples stored for 10 days at ambient temperature or -20°C [24]. This indicates that an adapted version of the WBA with dry stimulated plasma spots instead of frozen supernatants could be developed for the rural less-equipped health centers, thereby avoiding a cold-chain transport and decreasing the cost of transport to reference centers. Despite a loss in quantity, the ratio between D0 and EOT remained identical by which a pretest-posttest would be advised. In addition, no significant differences in cytokine/chemokine expressions between SLA from *L. infantum* and SLA from *L. donovani* stimulation were observed. Similar results were reported in studies determining the CMI at endemic areas for *L. infantum* and *L. donovani* [21]. This suggests that many immunodominant antigens are shared across the species and a global assay could be developed [40].

Despite a number of inherent and important limitations such as the lack of treatment failure cases, low number of individuals, and lack of long-term follow-up after treatment stop, we believe these findings can spur and steer larger cohort studies to evaluate its diagnostic and prognostic value as a treatment monitoring tool.

## 5. Conclusion

This pilot study indicated a detectable restoration of cell-mediated immunity against VL that could be used to follow up treatment efficacy and enable better patient management. This longitudinal pilot study showed that IFN-*γ* had a steady and on average 13-fold increase from time of diagnosis until end of treatment in successfully treated VL patients and would be the recommended analyte out of a panel of 7 previously proposed markers to be prioritized. We hope the recommendations in this work on the use of WBA in combination with filter paper will facilitate treatment monitoring studies of more VL patients in the search for an alternative less-invasive test-of-cure. In particular, an early marker of treatment efficacy would be highly warranted to limit the duration or steer the chemotherapeutic choice of treatment.

## Figures and Tables

**Table 1:** Sociodemographic and clinical characteristics of study participants in Gondar, Ethiopia.

Variables		VL patients (*n* = 13)
Demographic characteristics		
Male gender, *n* (%)		13 (100)
Age (years), median (min–max)		22 (17-27)
Occupation, *n* (%)		
Farmer		6 (46.1)
Daily labourer		7 (53.9)
Literacy, *n* (%)		5 (38.4)
Marital status, *n* (%)		
Single		12 (92.3)
Married		1 (7.7)
Migrant worker, *n* (%)		12 (92.3)
Clinical characteristics		
BMI, median (kg/m^2^) (min–max)		16.4 (13.6-18.4)
Fever, *n* (%)		9 (69.2)
Conjunctival pallor, *n* (%)		12 (92.3)
Edema, *n* (%)		6 (46.2)
Other pathology, *n* (%)		3 (23.1)
Palpable spleen size (cm), mean ± SD		9.1 ± 4.8
Parasite load (parasites/field), median (min–max)		3 (1-6)
Type of treatment, *n* (%)	PM+SSG	10 (76.9)
	AmBisome	3 (38.5)
Treatment duration (days), median (min–max)	PM+SSG	17 (15-20)
	AmBisome	19 (14-22)

min: minimum; max: maximum; *n*: total number; SD: standard deviation; PM: paromomycin; SSG: sodium stibogluconate.

**Table 2:** Proportion of patients that produced levels above the calculated optimal cut-off values after SLA stimulation of a whole blood sample.

Analytes (cut-off value)	*L. donovani*			*L. infantum*		
	D0 *N* (%)	W1 *N* (%)	EOT *N* (%)	D0 *N* (%)	W1 *N* (%)	EOT *N* (%)
IFN-*γ* (48.1 pg/ml)	4/13 (31)	10/13 (77)	13/13 (100)	4/13 (31)	10/13 (77)	13/13 (100)
TNF-*α* (25.4 pg/ml)	4/13 (31)	9/13 (69)	10/13 (77)	4/13 (31)	10/13 (77)	11/13 (85)
IP-10 (452.9 pg/ml)	3/13 (23)	11/13 (85)	12/13 (92)	3/13 (23)	9/13 (69)	12/13 (92)
IL-2 (20.0 pg/ml)	0/13 (0)	0/13 (0)	5/13 (38)	0/13 (0)	0/13 (0)	5/13 (38)
IL-10 (37.3 pg/ml)	5/13 (38)	5/13 (38)	4/13 (31)	3/13 (23)	5/13 (38)	5/13 (38)

D0: active moment, day zero; W1: first week of the treatment; EOT: end of treatment; *N*: number of patients; %: percentage of positives patients that produce cytokines/chemokines.

## Data Availability

The data used to support the findings of this study are restricted by the research ethics committee of the School of Biomedical and Laboratory Sciences, College of Medicine and Health Sciences, University of Gondar, and the Institutional Review Board of the Institute of Tropical Medicine in order to protect patient privacy. Data are available from Wim Adriaensen for researchers who meet the criteria for access to confidential data.

## Supplementary Materials

Supplementary Materials Supplementary Table 1 showed the levels of cytokines and chemokines produced after PHA stimulation whole blood from VL patients in the active moment, during the treatment and the end of treatment. In the Supplementary Figure 1, we showed the fold change in each cytokine/chemokine concentration after one week of treatment (W1) and at the end of the treatment (EOT), in relation to their concentration at the time of active disease (D0) after SLA stimulation from *L. infantum*.
