# Supplementary material for: Whole Blood Stimulation Assay as a Treatment Outcome Monitoring Tool for VL Patients in Ethiopia: A Pilot Evaluation
Source: J Immunol Res. 2020 Jan 23;2020:8385672. doi: 10.1155/2020/8385672 (PMC7193677; doi:10.1155/2020/8385672)
Supplement: Supplementary Materials — Supplementary Table 1 showed the levels of cytokines and chemokines produced after PHA stimulation whole blood from VL patients in the active moment, during the treatment and the end of treatment. In the Supplementary Figure 1, we showed the fold change in each cytokine/chemokine concentration after one week of treatment (W1) and at the end of the treatment (EOT), in relation to their concentration at the time of active disease (D0) after SLA stimulation from L. infantum. [file 8385672.f1.pdf]

Supplementary Material

Supplementary Table 1 showed the levels of cytokines and chemokines produced after PHA-stimulation whole blood from VL patients in the active moment, during the treatment and the end of treatment.

Supplementary table 1. Median concentration (pg/ml) (IQR) of cytokine/chemokine after PHA stimulation of 13 VL patients from Gondar, Ethiopia.

Cytokine/ Chemokine	D0	W1	EOT
IFN-γ	0.1 (0-5.54)	2.52 (0.39-3.71)	3.39 (1.56-15-27)[b*]
TNF-α	8.03 (5.19-19-74)	62.00 (8.81-214.3)[a**]	206.50 (114.9-726.7) [b****, c**]
IP-10	5.19 (0-73.10)	34.26 (1.53-112.90)	122.10 (39.70-842.10)[b**]

[a] D0 *vs* W1
[b] D0 *vs* EOT
[c] W1 *vs* EOT
D0: active moment of the disease; W1: one week during the treatment; EOT: end of treatment. *p<0.05; **p<0.01; ****p<0.0001.

In the supplementary Figure 1, we showed the fold change in each cytokine/chemokine concentration after one week of treatment (W1) and at the end of the treatment (EOT), in relation to their concentration at the time of active disease (D0) after SLA-stimulation from *L. infantum*.

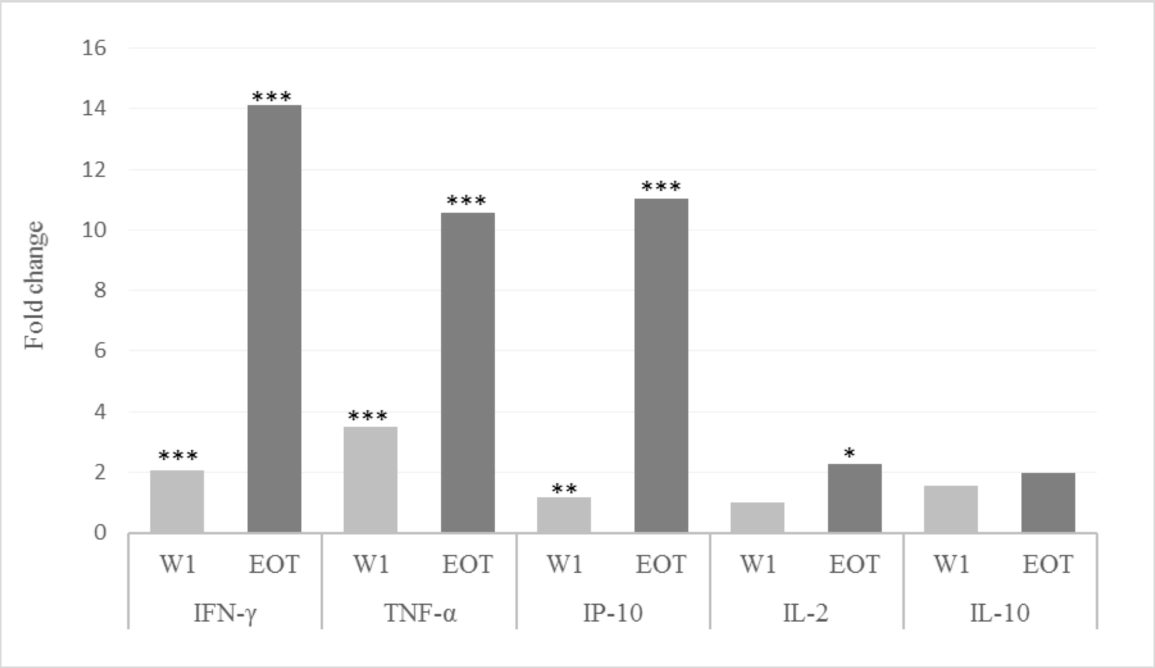

Supplementary figure 1. Fold changes in cytokine/chemokine concentrations during treatment of 13 VL patients, measured in soluble *L. infantum* antigen stimulated plasma. Fold increase during W1 and EOT compared to D0 was calculated by dividing the value of (W1, EOT) by D0 values. P values are represented for comparison with time of diagnosis (D0). *p<0.05; **p<0.01; ***p<0.001.
